# Additive manufacturing of hollow connected networks for solar photo-Fenton-like catalysis†

**DOI:** 10.1039/d4su00312h

**Published:** 2024-10-16

**Authors:** Miguel Ángel Gracia-Pinilla, Norma Alicia Ramos-Delgado, Cristian Rosero-Arias, Remco Sanders, Stephan Bartling, Jędrzej Winczewski, Han Gardeniers, Arturo Susarrey-Arce

**Affiliations:** a Department of Chemical Engineering, MESA+ Institute, University of Twente P. O. Box 217 Enschede 7500AE The Netherlands; b Mesoscale Chemical Systems, MESA^+^ Institute, University of Twente PO Box 217 Enschede 7500 AE The Netherlands a.susarreyarce@utwente.nl; c Facultad de Ciencias Físico Matemáticas, Universidad Autónoma de Nuevo León San Nicolás de los Garza Nuevo León 66455 Mexico miguel.graciapl@uanl.edu.mx; d Centro de Investigación e Innovación Tecnológica, IxM CONAHCyT-Tecnológico Nacional de México/I.T. Nuevo León Apodaca Nuevo León Mexico norma.rd@nuevoleon.tecnm.mx; e School of Engineering and Sciences, Tecnologico de Monterrey Eugenio Garza Sada 2501 Monterrey 64849 NL Mexico; f Leibniz-Institut für Katalyse e.V. Albert-Einstein-Strasse 29a D-18059 Rostock Germany

## Abstract

A 3D-printing approach is used to fabricate green bodies/precursor microarchitectures that, upon annealing, allow the fabrication of hierarchical 3D hollow microarchitectures (3DHMs). The 3DHMs are composed mainly of TiO_2_ and inorganic stabilizers that enable the production of inorganic cellular units upon thermal annealing at 650 °C. Morphological inspection reveals that the 3D architecture beams comprise TiO_2_ nanoparticles (NPs). The inner and outer diameters of the hollow beams are ∼80 μm and ∼150 μm, retained throughout the 3D hollow network. A proof-of-concept photo-Fenton reaction is assessed. The 3DHMs are impregnated with α-Fe_2_O_3_ NPs to evaluate solar photo-Fenton degradation of organic compounds, such as MB used as control and acetaminophen, an organic pollutant. The optical, structural, and chemical environment characteristics, alongside scavenger analysis, generate insights into the proposed solar photo-Fenton degradation reaction over TiO_2_ 3DHMs loaded with α-Fe_2_O_3_. Our work demonstrates newly hollow printed microarchitecture with interconnected networks, which can help direct catalytic reactions.

## Abstract 2

Additive manufacturing of hollow interconnected networks for the degradation of pharmaceuticals.

## Graphical abstract

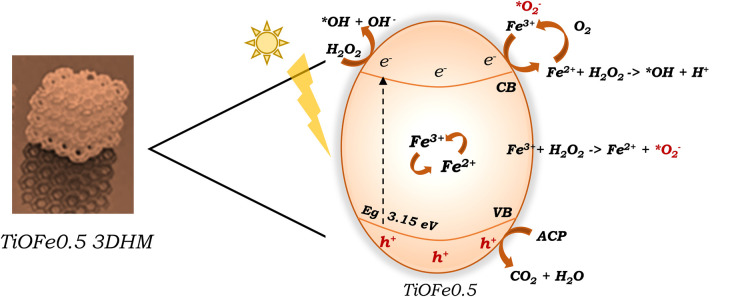

**Sustainability spotlight**

Additive manufacturing (AM), also known as 3D printing, can leverage the production potential of hierarchical 3D hollow microarchitectures (3DHMs) primarily composed of TiO_2_ impregnated with α-Fe_2_O_3_ to address the critical need for pollutant degradation in water at neutral pH. This approach enables efficient solar-driven photo-Fenton reactions to mineralize persistent organic pollutants, such as acetaminophen, improving water quality and promoting environmental health. By utilizing 3D printing, we advance the production of interconnected microarchitectures, which enhances photocatalytic reaction efficiency. This innovation aligns with the UN's Sustainable Development Goals (SDGs) 6 (Clean Water and Sanitation) and 12 (Responsible Consumption and Production), contributing to cleaner water systems and more sustainable industrial processes. Our research underscores the potential of AM in fostering sustainable solutions for global environmental challenges.

## 1. Introduction

Additive manufacturing (AM), also known as 3D printing, has gained significant attention recently due to its ability to fabricate complex geometries with high precision.^1^ While AM has been widely used for manufacturing polymers and metals, the application of this technology to fabricate ceramics is still in its infancy,^2–4^ particularly for environmental purposes.^5^ Several AM techniques have been employed for ceramics, for example, vat photopolymerization, powder bed fusion, binder jetting, material extrusion including electrospinning, and melt electrowriting, which arguably have been proposed as AM technologies.^1,6–8^ Among the latest AM approaches, vat photopolymerization includes stereolithography, two-photon lithography, selective laser sintering, and digital light processing, which employs photopolymer resins that can be cured upon light exposure.^9^

In two-photon photopolymerization, two photons are absorbed simultaneously by a photoinitiator molecule, initiating the polymerization reaction.^10^ The process allows for the fabrication of objects with sub-diffraction limited resolution and high spatial resolution.^11^ However, special considerations should be undertaken for such sub-diffraction resolution, particularly for photoresin-dispersed NPs.^11^ Large NPs can accelerate curing but might exacerbate uniformity due to energy variations. Such energy variations are related to refractive index mismatch between the photoresin components and NPs, affecting the resolution.^11^ Therefore, metal/metalloid organics or well-dispersed ultra-small NPs in the photoresins are more desirable for vat polymerization processes,^12–14^ facilitating the production of open intricated structures dedicated to photocatalysis.

3D-printing open intricated designs are often desired for light management and chemical reaction/diffusion control.^15,16^ Among the various 3D printing approaches, one-photon polymerization using digital light processing (DLP) can be achieved due to its rapid prototyping capabilities. Nevertheless, it should be highlighted that another printing method can achieve similar printing competencies to DLP. This is the case for stereolithography (SLA), where the main difference with DLP lies in the photoresin light-curing process. In this case, DLP cures an entire layer simultaneously, while SLA traces a path with the UV light laser curing along the traced path. From the latest printing developments, SLA or DLP are two critical technologies of great similitude for the AM of hierarchical inorganic designs. However, hereafter, DLP printing is our method of choice and is thus assessed further for producing new 3DHMs.

DLP printing relies on selectively embedding geometrical information spatially using a light source that induces the polymerization of a photoresist containing monomers and photoinitiators alongside some solvent traces. DPL permits rapid printing speeds and high accuracy within tens of micrometers resolution features, allowing the fabrication of intricated multiscaled microarchitectures of various materials components, such as polymer blends with ceramic NPs like Al_2_O_3_, SiO_2_, and TiO_2_ (hereafter pre-ceramic).^11^ Unlike other AM methods for producing pre-ceramic architectures (*e.g.*, injection molding, fused deposition modeling, and ink-jet printing), two-photon lithography (TPL) and direct laser writing (DLW) combined with atomic layer deposition (ALD) has enabled the production of hollow ceramic cellular microarchitectures upon the removal of a polymer template.^17,18^ However, DLP printing of 3DHMs using NPs has not been reported.

Compared to other ceramics used in 3D printing (SrZrO_3_, BaZrO_3_, CaZrO_3_, ZrO_2_ : Y_2_O_3_, ZrO_2_, SiO_2_, SnO_2_, or ZnO).^19–23^ TiO_2_ has demonstrated excellent functionality in photocatalysis. TiO_2_ is an n-type semiconductor that promotes electron–hole pair generation upon UV light exposure, enabling, for example, the chemical degradation of pollutants.^24^ However, an ideal architected photocatalyst should effectively promote the degradation of organic pollutants under visible light, *e.g.*, during the heterogeneous (solar) photo-Fenton process.^25^ The heterogeneous (solar) photo-Fenton degradation combines Fe and a semiconductor like TiO_2_. When the light reaches the TiO_2_, the photogenerated electrons in the conduction band are used to accelerate the Fenton redox reaction on the TiO_2_ surface by increasing the cycle rate of Fe^3+^/Fe^2+^, which promotes the decomposition of H_2_O_2_, yielding the formation of ˙OH radicals and other strongly oxidizing species.^26^ In this case, α-Fe_2_O_3_, can promote Fe^3+^/Fe^2+^ formation and improve a photo-Fenton process^27,28^ under natural solar light when coupled to TiO_2_. 3DHMs with α-Fe_2_O_3_ and TiO_2_ are expected to ease the use of DLP in chemical conversion and environmental remediation, which has not been much explored compared with other areas, such as energy storage.^5^ In this context, the development of TiO_2_-based 3D hollow microarchitectures (3DHMs) decorated with α-Fe_2_O_3_ NPs offers a promising approach to enhancing the photo-Fenton process, which can effectively degrade organic pollutants under natural solar light. Given the nature of pollutants like methylene blue (MB) and acetaminophen (ACP), commonly found in wastewater, using our advanced photocatalytic 3D printed materials becomes particularly relevant, which is why those were chosen as model pollutants.

MB and ACP have been extensively used in clinical and industrial settings, yet they present notable risks to human health and the environment. MB is primarily used in medicine to treat methemoglobinemia, as a surgical dye, and in antimicrobial photodynamic therapy. It is also employed as a staining agent in microbiology.^29^ Additionally, it has been explored for its potential antiviral properties, particularly in the context of COVID-19, although further clinical studies are needed to establish its efficacy.^30^ Furthermore, MB has been linked to neurotoxicity, including serotonin syndrome and oxidative stress, particularly at high doses or in sensitive populations.^31,32^ On the other hand, ACP, widely used for its analgesic and antipyretic properties, poses significant risks of hepatotoxicity and renal impairment when overdosed, and its chronic use can exacerbate these risks.^33^ The presence of ACP in wastewater contributes to environmental pollution, as it is not entirely removed during wastewater treatment. This poses risks to aquatic organisms and could potentially enter the human food chain.^34–36^ Environmentally, both MB and ACP are still detected in water bodies.^33,37^

Hence, this work introduces a DLP printing approach to fabricate TiO_2_ 3DHMs decorated with α-Fe_2_O_3_ NPs for the (solar) photo-Fenton process under neutral pH. The 3DHMs' optical, structural, and chemical characteristics, alongside scavenger analysis, generate insights into the photo-Fenton degradation reaction. The results open new horizons for eliciting interconnected network geometries within 3D microarchitecture for catalytic reactions.

## 2. Experimental section

### 2.1 3D photo-Fenton catalyst with hollow architecture

TiO_2_ 3DHMs involve the addition of 2 g of TiO_2_ P25 Evonik (particles with diameters between 9 and 53 nm) and 1 g of Al_2_O_3_ (particles with diameters lower than 50 nm) dispersed in 100 mL of Phrozen Aqua Gey 4K photoresin for digital light processing (DLP). The Phrozen Aqua Grey 4K photoresin contains bis-acyl phosphine oxide (BAPO) as a photoinitiator. The mixture was blended using a magnetic stirrer and heated to 40 °C during 4 h mixing. Once a homogeneous photoresin and NPs mixture is achieved, the 3D architecture is printed using a Phrozen Mini 4K 3D printer. The printed TiO_2_ 3DHMs were placed in an oven (LH 15/12 Nabertherm) and calcined in air. During annealing, a temperature ramp was used as follows: (i) 1 °C min^−1^ until reaching 350 °C and kept for 3 h to remove the organic constituents in the resin, and (ii) 0.5 °C min^−1^ until reaching 650 °C and kept for 3 h to remove the remnant carbon and ensure the anatase phase.

### 2.2 3D photo-Fenton catalyst loaded with α-Fe_2_O_3_

First, the α-Fe_2_O_3_ was obtained by calcination of iron(ii) oxalate dihydrate (FeC_2_O_4_·2H_2_O ≥ 99.0% Sigma-Aldrich) at 450 °C for 2 h with a heating rate of 2 °C. Then, the printed TiO_2_ 3DHMs were impregnated with the synthesized α-Fe_2_O_3_ by impregnation with a suspension of α-Fe_2_O_3_ and ethanol. Various α-Fe_2_O_3_ wt% loadings were used, *e.g.*, 0, 0.1, 0.25, 0.5, 1, and 2 wt%. After the loading was completed in ethanol, the TiO_2_ 3DHMs with α-Fe_2_O_3_ were treated at 60 °C for 8 hours to remove the excess ethanol, followed by heating at 200 °C using a ramp of 1 °C min^−1^ in an air atmosphere.

### 2.3 Characterization of the 3D photo-Fenton catalyst

#### Scanning electron microscopy and transmission electron microscopy

Scanning electron microscopy (SEM) and energy dispersive X-ray (EDX) were used to provide insight into the architecture network and composition. SEM-EDX images were acquired using a Carl Zeiss Merlin AURIGA CrossBeam workstation at 1.4 kV acceleration voltage, coupled with High-Efficiency Secondary Electron Detector (HE-SE2). The 3D TiO_2_ hollow architectures were broken before the SEM-EDX analysis and placed over carbon tape without further specimen preparation. The α-Fe_2_O_3_ was investigated with a transmission electron microscope (TEM) from FEI (Titan G2 80-300) set to an accelerating voltage of 300 kV.

#### X-ray diffraction

X-ray powder diffraction (XRD) of the 3D hollow architectures was carried out by grinding the structures until a powder was obtained. Then, the powders were deposited onto zero-diffraction substrates (Bruker) and scanned at a 2*θ* range of 20–80° using a benchtop X-ray powder diffractometer (D2 Phaser, Bruker) with a LynxEye detector and a Cu-Kα source operated at 30 kV and 10 mA.

#### X-ray photoelectron spectroscopy

The electrochemical environment at the surface of the pulverized TiO_2_ 3DHMs with α-Fe_2_O_3_ was analyzed with X-ray Photoelectron Spectroscopy (XPS). The measurements were performed on an ESCALAB 220iXL (Thermo Fisher Scientific) with monochromated Al Kα radiation (*E* = 1486.6 eV). Samples were prepared on a stainless-steel holder with conductive double-sided adhesive carbon tape. The measurements were performed with charge compensation using a flood electron system combining low-energy electrons and Ar^+^ ions (*p*_Ar_ = 1 × 10^−7^ mbar). The electron binding energies were referenced to the C 1s core level of carbon at 284.8 eV (C–C and C–H bonds). The peaks were deconvoluted with Gaussian–Lorentzian curves for quantitative analysis using the software Unifit 2023. The peak areas were normalized by the spectrometer's transmission function and Scofield's element-specific sensitivity factor.

#### Ultraviolet-visible spectroscopy

The optical characterization was performed with an ultraviolet (UV), visible (Vis), near-infrared (UV-Vis-NIR) spectrophotometer (PerkinElmer Lambda 950 UV-Vis-NIR) in the 250 to 800 nm range, employing an integrating sphere. The reflectance (*R*) spectra were collected separately from the band gap (*E*_g_) calculated using the Kubelka–Munk method. A linear region was used to extrapolate to the *x*-axis intercept to determine the *E*_g_ values of the various pulverized TiO_2_ hollow architectures loaded with and without α-Fe_2_O_3_.

#### Brunauer–Emmett–Teller

The Brunauer–Emmett–Teller (BET) specific surface area was obtained by measuring the N_2_ adsorption–desorption with a Bel-Japan Minisorp II analyzer. Before BET, the TiO_2_ hollow architecture loaded with and without α-Fe_2_O_3_ was treated under vacuum at 100 °C for 24 h.

#### Thermogravimetric analysis

The thermogravimetric analysis (TGA) was carried out by using a piece of a printed pre-ceramic architecture containing TiO_2_ (Fig. S2†) in a NETZSCH STA 449F3 high DTA (NETZSCH-Gerätebau GmbH, Seligenstadt, Germany) furnace at a temperature range of 40–1000 °C, at 5 °C min^−1^ ramp, in air.

### 2.4 Photoactivity of the photo-Fenton catalyst

A proof-of-concept photo-Fenton reaction was assessed. In this case, the degradation of methylene blue (MB) showcases the performance of the TiO_2_ 3DHMs with α-Fe_2_O_3_ as a heterogeneous photo-Fenton catalyst. All the heterogeneous photo-Fenton catalysts with or without α-Fe_2_O_3_ were tested at pH = 7. The experiments used 3 mL of MB solution (*ca.* 10 mg L^−1^). The 3D photo-Fenton catalyst (*ca.* 15 mg) was submerged in MB for 30 min in the dark to promote the adsorption–desorption equilibrium. Next, 3.5 μL of hydrogen peroxide (30% v/v; 10 mM) was added, and the reactor was illuminated with a white-LED lamp (white LED 420–700 nm, 60 mW, Instytut Fotonowy) placed at a perpendicular distance of 18 cm above the reactor. When the photo-Fenton reaction was initiated, aliquots were taken from the reactor over multiple intervals. The decomposition reaction was tracked using a UV-Vis spectrometer from Avantes AvaSpec-ULS2048CL-EVO-RS. The *λ* = 664 nm was used as it corresponds to the maximum absorbance of MB.

### 2.5 Reusability of the photo-Fenton catalyst

The most active 3D photo-Fenton catalyst loaded with α-Fe_2_O_3_ was tested at pH = 7 to evaluate the reusability for 3 consecutive cycles. Between each cycle, the photo-Fenton catalyst loaded with α-Fe_2_O_3_ was washed with isopropanol three times. After that, it was dried for 4 h at 60 °C. Iron leaching was measured using the HACH TPTZ method #8112 (range 0.012 to 1.80 mg L^−1^ of Fe).

### 2.6 Solar photo-Fenton

The solar photo-Fenton experiments were carried out using a batch reactor with 12 mL of acetaminophen solution (20 mg L^−1^) and 4 pieces of TiO_2_ 3DHMs with 0.5% α-Fe_2_O_3_, which floated on the surface of the system; no agitation was used. For the first 30 min the system was in darkness to allow equilibrium in adsorption–desorption processes, then it was taken outdoors to be exposed to natural sunlight, and the radiation was measured with a Delta OHM HD2102.2 radiometer (range: 315–400 nm). Samples were collected as needed, then filtered and immediately analyzed by High-Performance Liquid Chromatography (HPLC) in Agilent 1260 Infinity equipment, using a C18 column and at *λ* = 242 nm.

### 2.7 Scavengers

The scavenger test is assessed to investigate the mechanism involved in the solar photo-Fenton process of the TiO_2_ 3DHMs with 0.5% α-Fe_2_O_3_ in the acetaminophen degradation. The tests have been carried out during the degradation of acetaminophen with either 1-butanol (10 mM), AgNO_3_ (10 mM), benzoquinone (*p*-BZQ, 10 mM), and ethylenediaminetetraacetic acid (EDTA-2Na, 10 mM). 1-Butanol, AgNO_3_, *p*-BZQ, and EDTA were used as scavengers to capture hydroxyl radicals (˙OH), electrons (e^−^), superoxide radicals (˙O_2_^−^), and holes (h^+^), respectively.

## 3. Results and discussions

### 3.1 3D hollow microarchitectures

**Fig. 1:**
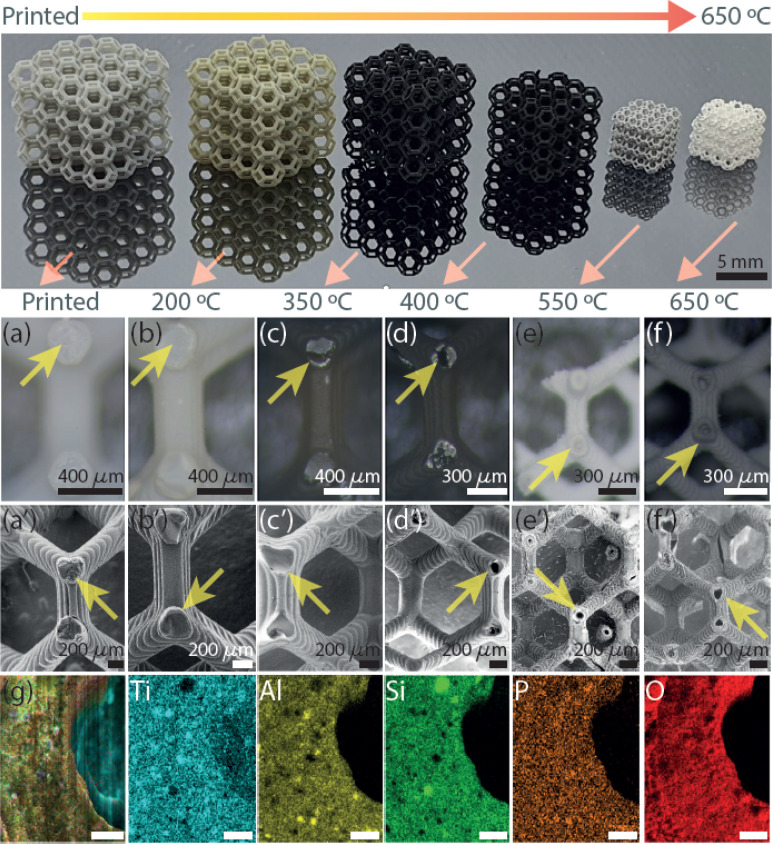
Top: smartphone image of thermally treated hollow 3D printed complex lattice microarchitectures with sub-millimeter features. An untreated printed structure followed by thermally treated lattices in the air until 650 °C is shown. Bright-field microscope images of lattice structure cross-section for the (a) printed and annealed lattices at (b) 200, (c) 350, (d) 400, (e) 550, and (f) 650 °C. SEM images of the (a′) printed and calcined structures at (b′) 200, (c′) 350, (d′) 400, (e′) 550, and (f′) 650 °C. The yellow arrow highlights the inside opening of the hollow 3D network. EDX-mapping of half-beam cross-section: (g) merged image, single channels from left to right are Ti, Al, Si, P, and O. Scale bars in (g) represent 100 μm.

The AM of the 3D microarchitecture with hollow connected networks starts using TiO_2_ NPs suspended with Al_2_O_3_ NPs in the photoresin-containing photoinitiator suitable for DLP printing. The reason for the use of Al_2_O_3_ NPs is to increase the hollow structure stability in the TiO_2_ 3DHMs. It should be noted that Al_2_O_3_ can also be printed alone (Fig. S1†). The absence of Al_2_O_3_ NPs yields brittle ceramic microarchitectures. The printed 3DHMs with TiO_2_ and Al_2_O_3_ NPs are displayed in Fig. 1. At the top row of Fig. 1, 3DHMs are shown as printed and annealed over various temperatures from 200 °C to a maximum of 650 °C. A closer look using a bright-field microscope and SEM image of the printed structure is shown in Fig. 1a and a′. Bright-field microscope and SEM images of the structures annealed at 200 °C are shown in Fig. 1b and b′. No evidence of hollow characteristics has been found at 200 °C. However, the color of the microarchitectures changes from grey-yellow to black upon annealing in air at 350 °C. For such a temperature, bright-field microscope and SEM images are shown in Fig. 1c and c′. When inspecting with a bright-field microscope, small cracks are found over the black microarchitecture. The cracks are transformed into small apertures at 400 °C, as revealed in Fig. 1d and d′. Compared to the 3D printed structure annealed at 350 °C, a size reduction of ∼20% for the 3D microarchitecture annealed at 400 °C is found. Increasing the annealing treatment to 550 °C makes the 3DHMs' coloration vary to light white-grey (Fig. 1e). An additional 30% size reduction is observed in Fig. 1e and e′. The 3DHM prevails up to 650 °C (Fig. 1f and f′). Aside from the white color in Fig. 1f and f′, no significant morphological changes are observed. The results reveal that the TiO_2_ 3DHMs are formed with tensile stress values close to 10 MPa ± 2 MPa. This indicates that the mechanical strength of the structures is relatively low and assigned to the hollow network character. However, the mechanical stability should not limit their applications in heterogeneous solar photo-Fenton. Additionally, it should be noted that the selected temperatures in Fig. 1 follow the temperature profile obtained using TGA (Fig. S2†). To this end, elemental analysis of the 3D hollow architecture annealed at 650 °C is shown in Fig. 1g merged image. Single channels reveal the presence of Ti (blue), Al (yellow), Si (green), P (brown), and O (red). From EDX, the Ti (12.6 at%) and Al (8.4 at%) correspond to the TiO_2_ and Al_2_O_3_ NPs. Si (9.6 at%) and P (1.2 at%) are related to the photoresin and photoinitiator, respectively. C and O are close to 8.2 at% and 59.8 at% and linked to the inorganic/organic compounds after annealing.

**Fig. 2:**
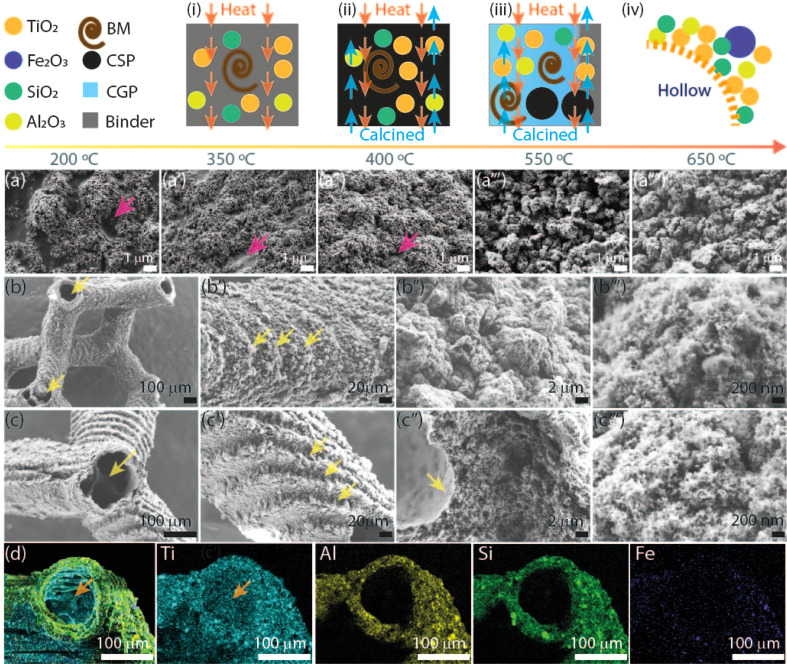
(i–iv) Schematic representation of the 3D hollow network. SEM image of the beam surface of a hollow 3D printed lattice structure. The pink arrows highlight the contrast differences between TiO_2_ and carbon-rich materials. Thermally treated lattices in the air at (a) 200 °C, (a′) 350 °C, (a′′) 400 °C (a′′′) 550 °C, (a′′′′) 650 °C. SEM image of the TiO_2_ hollow lattice loaded with (b–b′′′) 2 wt% and (c–c′′′) 0.25 wt% α-Fe_2_O_3_. Yellow arrows highlight lattice structure openings and geometrical features. EDX-mapping of half-beam cross-section containing 2 wt% α-Fe_2_O_3_: (d) merged image, single channels from left to right are Ti, Al, Si, and Fe.

SEM images of the TiO_2_ 3DHMs acquired over various temperatures are shown in Fig. 2a–a′′′′ to generate insights into the potential mechanism of the hollow structure formation. In Fig. 2a–a′′′′, SEM images are recorded over broken structure beams. Over the beams, darker and brighter contrasts are observed. Contrast differences are related to TiO_2_ (Al_2_O_3_ and SiO_2_) NPs and 3D-printed photoresin, as shown in Fig. 2a. Pink arrows highlight open darker areas, which can be associated with the organic compounds from the photoresist after annealing at 200 °C. As the temperature increases from 350 °C (Fig. 2a′) to 450 °C (Fig. 2a′′), the open areas disappear, and the NPs are more compact, visible at 550 °C (Fig. 2a′′′) and 650 °C (Fig. 2a′′′′). From the results in Fig. 1 and 2a–a′′′′, a mechanism for the hollow features in the 3DHMs is proposed in Fig. 2(i)–(iv). In this case, the printed microarchitecture (*i.e.*, green body) includes monomers, ceramic particles (*e.g.*, TiO_2_ and Al_2_O_3_), and cured photoresin. The hollow formation process might start at a low temperature with the binder melt (BM) in Fig. 2(i). Then, as the temperature increases, gaseous products decompose from the microarchitecture body. The effect becomes dramatic with the increase in calcined solid products (CSP), forming carbonized species around 400 °C (Fig. 2(iii)). At this stage, NPs start reaching the surface of the microstructure body. An increase in temperature (550 °C) leads to CSP forming calcined gas products (CGP) (Fig. 2(iii)). During CGP formation, NPs agglomerates retain a close packing. The remaining products decompose at higher temperatures, *i.e.*, 650 °C (Fig. 2(iv)), leading to the final 3DHMs with a multiscale network. Note that α-Fe_2_O_3_ is loaded after annealing.

The final TiO_2_ 3DHMs have a beam size of ∼230 μm, inner/outer diameter of ∼80 μm/150 μm, and a surface area (SA) of 82 m^2^ g^−1^, which remains similar even after impregnation with 2 wt% α-Fe_2_O_3_ (∼80 m^2^ g^−1^). The BET results reveal that α-Fe_2_O_3_ does not affect the SA or contribute to evident morphological changes over the TiO_2_ 3DHM, as shown for 2 wt% and 0.25 wt% loads in Fig. 2b–b′′′ and c–c′′′. Interestingly, it has been found that the microarchitecture is composed of NPs aggregates (Fig. 2b′′, b′′′, c′′ and c′′′) with tiny pores of an average of 9.4 nm as measured with BET. TiO_2_ 3DHM SA measured can then be contrasted to TiO_2_ NPs precursor with *ca.* SA of 57 m^2^ g^−1^. Although the results might suggest that 3DHMs increase the SA, it cannot be solely attributed to the 3DHMs network since Al_2_O_3_ NPs with SA of 115 m^2^ g^−1^ might contribute to the SA gain. Another observation is the features formed during layer-by-layer printing, which prevailed after annealing (Fig. 2b′ and c′). EDX merged mapping (Ti, Al, Si, and Fe) of the half-beam cross-section is shown in Fig. 2d. Next to Fig. 2d, Ti, Al, Si, and Fe single channels are also shown and used to compose the merged image. It is important to mention that Fe content measured with EDX < 2 wt% α-Fe_2_O_3_ is not observed. The TiO_2_ 3DHMs' complete XPS core spectra, including Fe 2p, Ti 2p, O 1s, and P 2p, are presented in Fig. S3.†

### 3.2 3D TiO_2_ hollow microarchitectures loaded with α-Fe_2_O_3_

Various 3DHMs loaded with α-Fe_2_O_3_ have been prepared using a wet impregnation. The set includes impregnated 3DHMs containing 2 (TiOFe2), 1 (TiOFe1), 0.5 (TiOFe0.5), and 0.25 (TiOFe0.25) wt% of α-Fe_2_O_3_. Additionally, three controls are used during the characterization steps. These are α-Fe_2_O_3_ as-synthesized, commercial TiO_2_, and 3DHMs of TiO_2_. It should be noted that for the 3DHMs loaded with α-Fe_2_O_3_, Fe has not been resolved with XPS (Fig. S3†). A potential reason for not observing Fe with XPS is that α-Fe_2_O_3_ can be allocated at ∼4 nm depth in the TiO_2_; hence, no clear XPS signal has been found. Compared to EDX, EDX is expected to have a larger penetration depth than XPS. EDX shows that it is still challenging to detect Fe even at α-Fe_2_O_3_ content than 2 wt% (Fig. 2d). From the latest results, we proceed to the morphological, structural, and chemical environment of the synthesized α-Fe_2_O_3_ only.

**Fig. 3:**
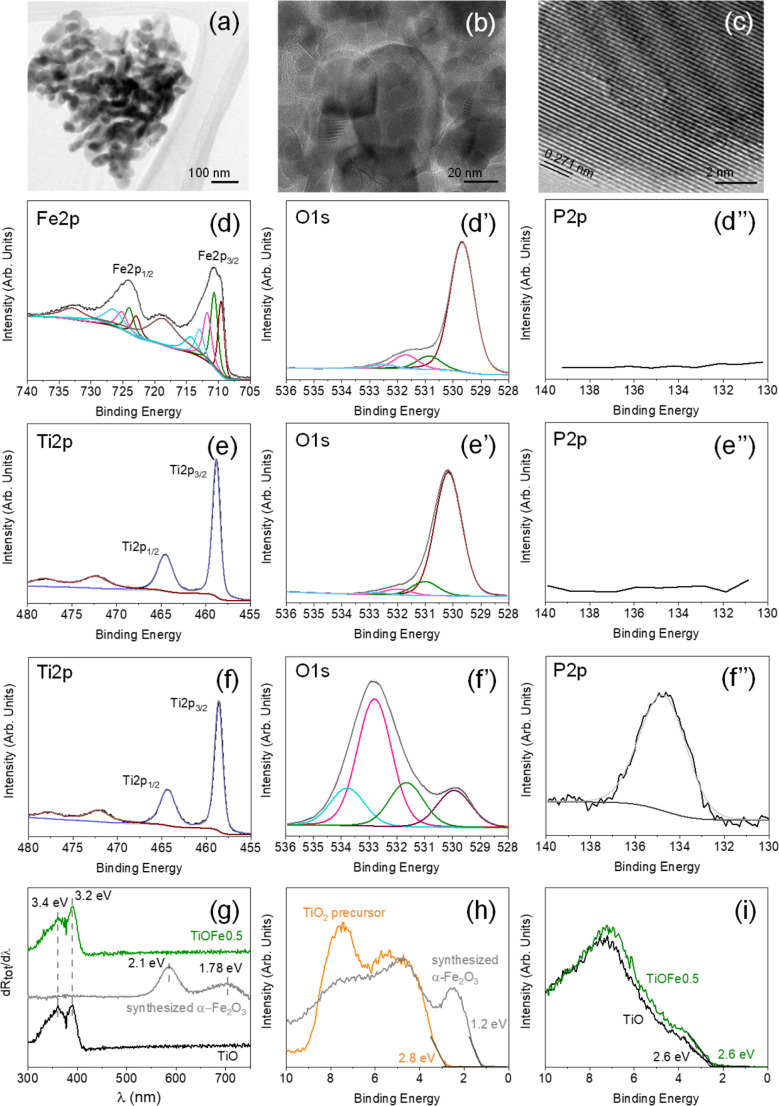
(a)–(c) TEM images of the synthesized α-Fe_2_O_3_. XPS core spectrum of (d) Fe 2p, (d′) O 1s, and (d′′) P 2p for the synthesized α-Fe_2_O_3_. XPS core spectrum of (e and f) Ti 2p, (e′ and f′) O 1s, and (e′′ and f′′) P 2p in (e and e′′) TiO_2_ precursor and (f and f′′) TiO_2_ 3DHMs loaded with 2 wt% α-Fe_2_O_3_. (g) The first derivative reflectance spectrum for TiO_2_ 3DHMs (black), synthesized α-Fe_2_O_3_ (grey), and TiO_2_ 3DHMs loaded with 0.5 wt% of α-Fe_2_O_3_ (green). Valence band determination for (h) TiO_2_ precursor (orange) and synthesized α-Fe_2_O_3_ (grey) and (i) TiO_2_ 3DHM (black) and TiO_2_ 3DHM loaded with 0.5 wt% α-Fe_2_O_3_ (green).

Our analysis starts with the use of TEM to understand the morphological and structural characteristics of the synthesized α-Fe_2_O_3_ loaded over the 3DHMs. The synthesized α-Fe_2_O_3_ comprises several grain types of about 100 nm or lower diameter that vary from rod-like to particle-like geometries (Fig. 3a and b). Fig. 3c shows the crystal lattice distance of 0.271 nm associated with planes (104) in α-Fe_2_O_3_ (ref. 38) TEM lattice distance from α-Fe_2_O_3_ agrees with the XRD pattern in Fig. S4a,† where (104) plane assignment has been confirmed for one of the most intense diffraction peaks. Unlike 3DHM TiOFe2, no α-Fe_2_O_3_ XRD diffraction peak is found (Fig. S4b†). Only TiO_2_ has been identified with XRD in Fig. S4b.†

Next, the chemical environment of the freshly synthesized α-Fe_2_O_3_ is assessed in Fig. 3d–d′′. XPS spectra for Fe 2p show two prominent peaks at around 710.7 and 724.5 eV (Fig. 3d) assigned to Fe 2p_3/2_ and Fe 2p_1/2,_ with their respective satellite peaks at 718.8 and 733 eV suggesting the presence of Fe^3+^ species from α-Fe_2_O_3_.^39^ The high-resolution XPS O 1s core level spectra exhibit a dominant contribution at *ca.* 529.7 eV attributed to lattice O in α-Fe_2_O_3_ (Fig. 3d′).^40^ Additional contributions ascribed to surface-adsorbed oxygen, H_2_O, OH groups, and/or organic compounds from air exposure are also detected at higher binding energies (531–533 eV).^41,42^

The XPS analysis of the freshly synthesized α-Fe_2_O_3_ is followed by the XPS analysis of the TiO_2_ precursor used to print the 3DHMs. The TiO_2_ XPS spectra show the presence of Ti 2p, O 1s, and P 2p in Fig. 3e–e′′. The XPS spectrum for Ti 2p (Fig. 3e) displays two prominent peaks at 458.5 and 464.3 eV. The peaks are assigned to Ti^4+^ from TiO_2_.^43^ XPS O 1s core level spectra exhibit the contribution at *ca.* 530.2 eV attributed to bulk O in α-TiO_2_ (Fig. 3e′).^44^ Surface-adsorbed H_2_O and –OH contributions are detected at higher binding energies (531–533 eV),^44^ but the presence of carbon should not be disregarded as some overlap might exist with the O 1s. As expected for the TiO_2_ precursor, no P has been found in Fig. 3e′′.

The XPS results from the synthesized α-Fe_2_O_3_ and TiO_2_ precursor are compared with TiO_2_ 3DHMs loaded with α-Fe_2_O_3_ 2 wt%. For this case, Ti 2p, O 1s, and P 2p are shown in Fig. 3f and f′′. The results reveal that the Ti 2p XPS core spectrum for Fig. 3f has features similar to Fig. 3e. The O 1s suggest the presence of other oxygen species (Fig. 3e′) from the TiO_2_ lattice (530 eV) and the contribution of H_2_O and ^−^OH but perhaps most prominently from carbon species associated with the carbonized photoresin and photoinitiator (531–534 eV).^44,45^ Interestingly, the P signature from the BAPO photoinitiator has been found in Fig. 3e′′, in which the P/Ti ratio remains close to 0.7 ± 0.1 for all ceramic microarchitectures. Compared to synthesized α-Fe_2_O_3_ and TiO_2_, all TiO_2_ 3DHMs loaded (also unloaded) with α-Fe_2_O_3_ show P. Overall, the results suggest that oxidized phosphorus species^45,46^ do not alter the bandgap (*E*_g_), as shown in Fig. S5.† TiOFe0.5 after reaction results are shown in Fig. 3g and compared with TiO and synthesized α-Fe_2_O_3_. The first derivative reflectance spectrum (Fig. 3g) agrees with the first derivative reflectance spectrum from A. Trenczek-Zajac *et al.*,^47^*ca. E*_g_ around 3.4 eV for TiO_2_ anatase phase. The authors also observed an additional *E*_g_ close to 3.2 eV attributed to the rutile phase, as we found in XRD for TiO_2_ (Fig. S4b†). There is no optical evidence for α-Fe_2_O_3_ in the 3DHMs microarchitectures maintaining similar optical characteristics after reaction than TiO_2_ (Fig. 3g and S5†).

**Fig. 4:**
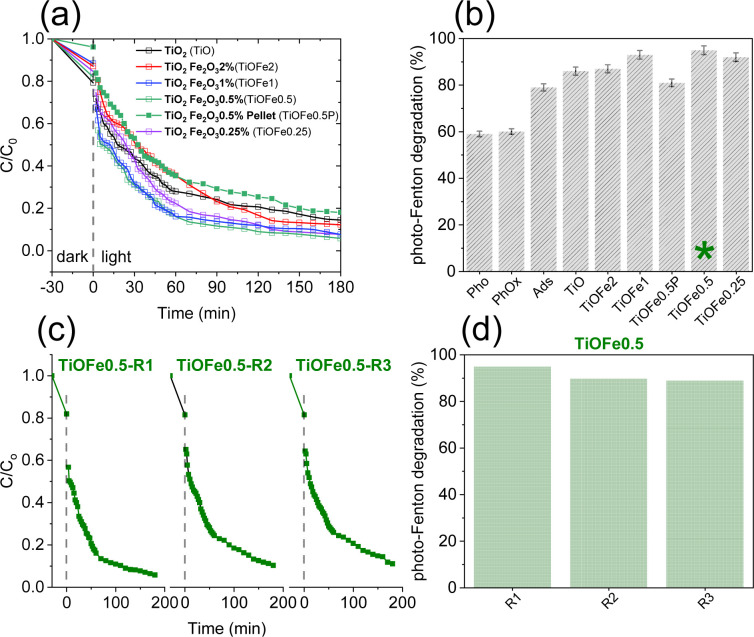
(a) MB degradation as a function of time for 3D microarchitectures composed of TiO_2_ and TiO_2_ loaded with 2, 1, 0.5, 0.25 wt% of α-Fe_2_O_3_. (b) Photo-Fenton degradation of MB for varied 3DHMs compositions. Controls include photolysis (Pho), photolysis in the presence of H_2_O_2_ (PhOx), adsorption of MB in the dark (Ads), and a TiO_2_ structure shaped as tabled without cellular geometries loaded with 0.5 wt% of α-Fe_2_O_3_ (TiOFe0.5P). (c) MB degradation as a function of time for TiOFe0.5. The 3DHM has been reused (R) three times (R1, R2, and R3). (d) Photo-Fenton reusability efficiencies using TiOFe0.5.

The optical transitions for α-Fe_2_O_3_ (Fig. 3g) are expected to be around 2.1 and 1.78 eV in the TiO_2_ 3DHMs with α-Fe_2_O_3_. Although optical transition variations are not observed, we separately analyze the valence band (VB) with XPS for TiO_2_ precursor and synthesized α-Fe_2_O_3_ in Fig. 3h. Additionally, TiO_2_ 3DHMs (TiO) and TiOFe05 are shown in Fig. 3i. Fig. 3h and i reveal that the TiO_2_ precursor and synthesized α-Fe_2_O_3,_ along with TiO and TiOFe0.5, have a VB of 2.8, 1.2 eV, 2.6, and 2.6 eV, respectively. It should be mentioned that the VB values remain close to 2.6 eV for 3DHMs microarchitectures loaded with 2, 1, 0.5, and 0.25 wt% α-Fe_2_O_3_. This includes TiOFe0.5 after the photo-Fenton reaction (Fig. 4) with a *ca.* VB of 2.6 eV. We then used the collected information to elaborate further on the energy band diagram from the *E*_g_ and VB results in Fig. S6.†

### 3.3 MB degradation functionality of the 3DHMs loaded with α-Fe_2_O_3_ during photo-Fenton reaction

The photocatalytic degradation of MB, a cationic dye, is assessed as a proof-of-concept reaction to select a suited outperforming 3DHMs with α-Fe_2_O_3_ for solar photo-Fenton reaction. The 3DHMs pre-selection includes TiO_2_ loaded with α-Fe_2_O_3_, *i.e.*, TiOFe2, TiOFe1, TiOFe0.5, and TiOFe0.25 (Fig. 4). The photo-Fenton reaction under controlled illumination for the 3DHMs in Fig. 4a shows the degradation of MB as a function of time. Among the α-Fe_2_O_3_ loaded 3DHMs, TiOFe1 and TiOFe0.5 have the highest MB degradation with fast degradation decay within the first 60 min, gradually decreasing until reaching 180 min. The degradation results are summarized in Fig. 4b, with TiOFe1 and TiOFe0.5 having the highest degradation, close to 93% and 95%. Fe leaching has been assessed for the structures. No Fe has been detected unless it is below the limit of detection of the colorimetric technique (0.012 mg L^−1^). The results suggest that the MB degradation is not due to Fe^3+^/Fe^2+^ in the solution but reactive species at the catalyst surface due to the loaded α-Fe_2_O_3_ serving as a photo-Fenton heterogeneous catalyst.

Other 3DHMs show an inferior MB degradation performance, *e.g.*, TiOFe2 with a degradation efficiency of 87%, which is quite comparable to TiO_2_ with 86% degradation. Interestingly, the TiO_2_-printed structures outperform a printed pellet that lacks cellular design, demonstrating the advantages of open cellular designs for photo-Fenton reactions (Fig. 4b). In this case, the printed pellet comprises the same TiO_2_ precursor and contains 0.5 wt% of α-Fe_2_O_3_ (TiOFe0.5P), which has shown the highest degradation in the form of 3DHMs. Furthermore, two additional controls have been carried out to demonstrate the performance of the TiO_2_ 3DHMs loaded with α-Fe_2_O_3_. In this case, control photooxidation (photolysis, Pho) without α-Fe_2_O_3_ leads to 59% MB degradation, while photooxidation with peroxide (PhoOx) without α-Fe_2_O_3_ shows a 60% MB degradation lower than 3DHMs TiOFe0.5. However, we should not disregard the MB adsorption capacity of the TiO_2_ 3DHMs in the dark (Ads), *ca.* 79% (Fig. 4b). Such large adsorption capacity is attributed to the hollow geometry of our microarchitectures with an estimated SA of 82 m^2^ g^−1^. From the adsorption results, increased photo-Fenton degradation activity for the 3DHMs loaded with α-Fe_2_O_3_ can additionally be related to an adsorption-degradation synergistic effect.^48^ This synergistic process can be explained as follows: during the first 30 min, before irradiation with white light, the 3DHMs with TiO_2_ and TiOFe0.5 adsorbed a higher concentration of MB, approximately 20% each. The rest of the materials adsorbed between 10 and 15% MB. However, after 30 min of white light, 3DHMs TiO_2_ removed 45% MB, and 3DHMs TiOFe0.5 removed 70% MB (Fig. S7†). This can be attributed to a synergistic effect between TiO_2_, which by itself has a good performance in MB adsorption, and α-Fe_2_O_3_, which helps to accelerate the degradation process by surface generation of oxidizing species.

The photo-Fenton results in Fig. 4a and b stress the importance of cellular ceramics architectures in photo-Fenton catalysis, which enables light penetration and, thus, enhances light harvesting throughout the open hollow geometry (Fig. 1 and 2). Printed TiO_2_ microarchitectures without hollow designs have been studied to the finest detail for water contaminants' degradation. However, direct ink-writing has been used.^49^ Other results for printed polymer structures coated with TiO_2_ have also demonstrated promising results for photodegradation.^24,50^ Altogether, the results are encouraging, as light penetration is bulk-limited in catalytic systems relying on photocatalytic beds. However, those can be designed as hollow open 3D cellular systems (Fig. 1 and 2) to drive chemical reactions at mild conditions, as demonstrated by our results during photo-Fenton degradation of pollutants like MB using neutral pHs (bulk pH = 7). To this end, we should not disregard the usability of our 3DHMs. Hence, in Fig. 4c and d, we reuse the most promising printed photo-Fenton catalyst, *i.e.*, TiOFe0.5. In this case, three reuse cycles, *i.e.*, TiOFe0.5-R1, TiOFe0.5-R2, and TiOFe0.5-R3, have been carried out. The results demonstrate that MB degradation is maintained close to 90% even after the third cycle. The results are promising as additive manufacturing approaches offering optical strategies like DPL to print 3DHMs are much desired in chemical conversion and environmental remediation, particularly at neutral pH.^26,51^ Environmental remediation functionality is then demonstrated during the photo-Fenton reaction under natural light illumination for the outperforming 3D hollow microarchitecture in Fig. 4 (*i.e.*, TiOFe0.5).

Recent studies have shown notable differences in the performance of proposed TiO_2_ 3D hierarchical materials (3DHMs) compared to traditional 3D printing catalysts. For instance, Martin de Vidales and co-authors used a floating photocatalyst, demonstrating that such catalysts are promising due to their low cost, easy implantation, and environmental compatibility using 3D Fused Filament Fabrication (FFF).^52^ Similarly, Li *et al.* used 3D printing ink. The authors reported that modified TiO_2_ catalysts exhibited varying efficiencies; composite photocatalysts are portable, easily designed, and may be extended to various functional materials.^53^ Viskadourakis *et al.* used Fused Deposition Modeling (FDM), and found that polymeric nanocomposites consisting of polystyrene matrix and nanoparticles of TiO_2_ achieved approximately 98% degradation of methylene blue, suggesting that shown that the transition from flat to 3D architectures results in a significant increase of the photocatalytic ability.^54^ In contrast, Bansiddhi *et al.* developed TiO_2_/SiO_2_/polymer scaffolds successfully fabricated using a stereolithography technique (SLA). The authors provided insights into the primary mechanism for dye removal through adsorption, while the photodegradation process has not been fully optimized.^55^ Furthermore, Grandcolas *et al.* prepared 3D structures printed in polyamide by selective laser sintering (SLS) and deposited TiO_2_ nanoparticles on 3D printed polyamide open structures using an impregnation method. The results showed enhanced photocatalytic degradation of MB.^56^ The same group developed a simple 3D-printed photocatalytic membrane reactor using a membrane of titania nanofibers prepared by electrospinning.^57^ Finally, Cao *et al.* presented Fe_2_O_3_/TiO_2_ nanocomposites; the results of MB photodegradation measurements indicated that the photocatalytic activity could be enhanced effectively by the fabrication of Fe_2_O_3_–TiO_2_ nanocomposites *versus* the Fe_2_O_3_ and TiO_2_.^58^ Compared to previously cited work, ours results uses DLP. Our 3DHM shows superior performance in the photodegradation efficiency of MB (95%) at a short degradation time and requires a low power of 0.06 W. The results are significantly more energy efficient than other methods requiring much higher power lamps (such as the 125 W UV lamps used in FDM or even 500 W used in the nanoparticles method). Furthermore, the structure size (cube truncated octahedron of 5 × 5 × 5 mm) is smaller and more precisely than many other methods; this is important because we have achieved more controlled 3D structures that can lead to a more efficient surface area-to-volume ratio, enhancing photocatalytic activity without needing excess material. In short, the DLP method balances high photocatalytic efficiency, low energy consumption, and quick degradation time, making it a more efficient and sustainable option. For comparison, see Table S1.†

### 3.4 ACP degradation functionality of the 3DHM during solar photo-Fenton reaction

**Fig. 5:**
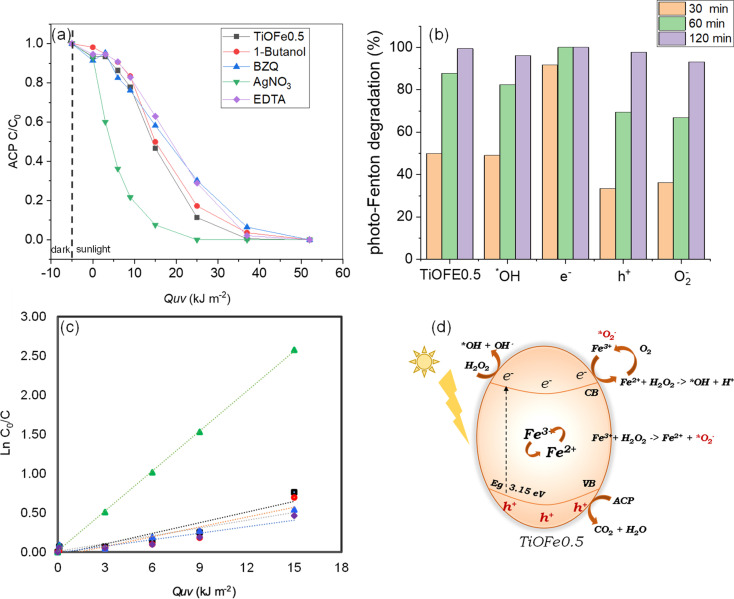
(a) ACP degradation as a function of accumulated energy (*Q*_UV_) without and for multiple scavengers like butanol, AgNO_3_, EDTA, and benzoquinone (˙OH, e^−^, h˙, and ˙O_2_^−^). (b) Solar photo-Fenton degradation of ACP for TiOFe0.5 3DHMs over time. (c) Kinetic of ACP degradation at 15 kJ m^−2^ using the Langmuir–Hinshelwood (L–H) kinetic model. (d) Proposed ACP degradation mechanisms during solar photo-Fenton reaction using TiOFE0.5 3DHM.

The functionality of the most active 3DHMs (*i.e.*, TiOFe0.5) is assessed for acetaminophen (ACP), an emerging organic pollutant that has come to the forefront of environmental issues.^59,60^ We also provide insights into the photo-Fenton degradation mechanism under natural solar light. For such purpose, the influence of radical scavengers on ACP solar photo-Fenton degradation is assessed, and the results are shown in Fig. 5a and b. Fig. 5a and b show the degradation of ACP as a function of solar accumulated energy *Q*_UV_ (kJ m^−2^) and ACP degradation over various time points for the TiOFe0.5 (no scavenger). In this case, the TiOFe0.5 achieves ACP degradation. In the same figures, scavengers, such as 1-butanol for ˙OH, AgNO_3_ for e^−^, EDTA for h^+^, and *p*-benzoquinone (*p*-BZQ) for ˙O_2_^−^ are used.^47,61^ The ACP *vs. Q*_UV_ plots show relatively fast decay within the first 10 kJ m^−2^ when 1-butanol, EDTA, and *p*-BZQ are used. After 10 kJ m^−2^, 1-butanol, EDTA, and *p*-BZQ follow a degradation trend, similar to TiOFe0.5, until 50 kJ m^−2^ (Fig. 5a). However, this is not the case for AgNO_3_, which has an abrupt overall trend. The role of AgNO_3_ has been proposed by Bansal *et al.*^62–64^ The authors evaluated the role of Ag in the Fe–TiO_2_ system during the photo-Fenton reaction. They proposed that Ag acts as an e-trap in two ways: (1) it promotes the Fenton reaction where Fe^2+^ is reduced to Fe^3+^ and, in the presence of H_2_O_2_, generates ˙OH radicals, and (2) the e^−^ reacts with surface O_2_ to yield the ˙O_2_^−^ radical which in turn reacts with H_2_O_2_ and generates ˙OH radicals. Both ways boost the oxidation process.

Therefore, we support our experimental results using the Langmuir–Hinshelwood (L–H) kinetic model (Fig. 5c). Fig. 5c and Table 1 demonstrate our attributions by estimating the reaction rates for the TiOFe0.5 without scavengers (*k*_app_ = 4.53 × 10^−2^) and 1-butanol (*k*_app_ = 4.17 × 10^−2^). However, this is not the case for EDTA and BZQ, whose degradation trend varies after 15 kJ m^−2^. Such variations are related to the capture of h^+^ and ˙O_2_^−^ by EDTA and BZQ during ACP degradation. In this case, h^+^ and ˙O_2_^−^ are important oxidant species, reflected by the drop in their reaction rates (Table 1). Interestingly, adding AgNO_3_ results in an abrupt enhancement of the reaction rate (*k*_app_ = 16.98 × 10^−2^), which implies the acceleration of e^−^ production and, thus, enhances degradation.^62–64^ The results demonstrate that ACP is fully degraded using our optimal TiOFe0.5 3DHMs. Additionally, Fig. 5a–c support that the ACP degradation mechanism is more prominently mediated by h^+^ and ˙O_2_^−^, while an e^−^ sequestrant can accelerate the pollutant degradation.

**Table 1:** Kinetic parameter of ACP degradation under natural solar photo-Fenton process, using the Langmuir–Hinshelwood (L–H) kinetic model

Scavenger	*k*_app_ (×10^−2^)	*R*
No scavenger	4.53	0.9357
1-Butanol	4.17	0.9214
Benzoquinone	3.30	0.9679
AgNO_3_	16.98	0.9996
EDTA	2.76	0.9507

From our photocatalytic results in Fig. 5a–c, we propose a reaction mechanism for ACP degradation that considers the synergy between TiO_2_ and α-Fe_2_O_3_. The proposed mechanism is shown in Fig. 5d and suggests photoelectron (e^−^) generation at the CB and holes (h^+^) at the VB. In this case, the e^−^ is moved to the TiO_2_ surface to reduce Fe^3+^ to Fe^2+^. This Fe^3+^/Fe^2+^ can lead to a redox cycle over the TiO_2_ surface and promotes the active generation of ˙OH in the presence of H_2_O_2_. Within this cycle, Fe^2+^ can react with oxygen O_2_ in the presence of protons (h^+^) to generate Fe^3+^, which can then produce ˙O_2_^−^ that participates in further cycles during the photo-Fenton degradation of reaction. It should be mentioned that the h^+^ in the VB does not contribute to the ˙OH generation by splitting the H_2_O molecule, from which it can be concluded that the degradation process is not drastically mediated by H_2_O splitting but by direct oxidation of the pollutant by the photogenerated h^+^. Therefore, it is proposed that h^+^ and ˙O_2_^−^ are the most important species that enable pollutant degradation.

## 4 Conclusions

This work reports a DPL printing approach that enables the production of TiO_2_ 3DHMs of varied α-Fe_2_O_3_ loadings. We demonstrate that our DPL approach is compatible with other ceramics, as showcased for Al_2_O_3_. TiO_2_ NPs mainly consolidate 3DHMs with cellular lattices with hollow networks. The functionality of the TiO_2_ 3DHMs is assessed during solar photo-Fenton degradation of ACP. For this step, synthesized α-Fe_2_O_3_ is loaded over the TiO_2_ 3DHMs. The optical, structural, and chemical environment characteristics, alongside scavenger analysis, generate insights into the photo-Fenton degradation reaction. The photo-Fenton results indicate that the 3DHMs with TiOFe0.5 composition exhibited the highest MB degradation efficiency, achieving nearly 95% removal within 180 minutes, underscoring the significant impact of α-Fe_2_O_3_ loading on photocatalytic activity. Additionally, the reusability tests revealed that the TiOFe0.5 structure maintained approximately 90% degradation efficiency even after three cycles, highlighting its stability and practicality for real-world applications. The unique open cellular architecture of the 3DHMs facilitated enhanced light penetration, contributing to the overall effectiveness of the photocatalytic process. Furthermore, the study provides insights into the degradation mechanisms involved, emphasizing the role of radical scavengers in the photo-Fenton reaction. These findings validate the use of 3D-printed TiO_2_-based materials in environmental remediation and pave the way for future research into optimizing photocatalytic systems for a broader range of pollutants. Integrating advanced manufacturing techniques with photocatalysis presents a promising avenue for addressing pressing environmental challenges, particularly in wastewater treatment.

## Data availability

Data are available upon request from the authors.

## Conflicts of interest

There are no conflicts to declare.

## Supplementary Material

SU-002-D4SU00312H-s001

## References

[cit1] Gibson I. , Rosen D. and Stucker B., Additive Manufacturing Technologies: 3D Printing, Rapid Prototyping, and Direct Digital Manufacturing, 2nd edn, 2015, pp. 1–498

[cit2] Zocca A., Colombo P., Gomes C. M., Günster J. (2015). J. Am. Ceram. Soc..

[cit3] Sun J., Ye D., Zou J., Chen X., Wang Y., Yuan J., Liang H., Qu H., Binner J., Bai J. (2023). J. Mater. Sci. Technol..

[cit4] Cramer C. L., Ionescu E., Graczyk-Zajac M., Nelson A. T., Katoh Y., Haslam J. J., Wondraczek L., Aguirre T. G., LeBlanc S., Wang H., Masoudi M., Tegeler E., Riedel R., Colombo P., Minary-Jolandan M. (2022). J. Eur. Ceram. Soc..

[cit5] Tan J. Z. Y., Ávila-López M. A., Jahanbakhsh A., Lu X., Bonilla-Cruz J., Lara-Ceniceros T. E., Andresen J. M., Maroto-Valer M. M. (2023). J. Mater. Chem. A.

[cit6] Xue J., Wu T., Dai Y., Xia Y. (2019). Chem. Rev..

[cit7] Tebyetekerwa M., Ramakrishna S. (2020). Matter.

[cit8] Robinson T. M., Hutmacher D. W., Dalton P. D. (2019). Adv. Funct. Mater..

[cit9] Yee D. W., Greer J. R. (2021). Polym. Int..

[cit10] Kawata S., Sun H. B., Tanaka T., Takada K. (2001). Nature.

[cit11] Winczewski J. P., Arriaga-Dávila J., Rosero-Arias C., Susarrey-Arce A. (2024). Trends Chem..

[cit12] Kotz F., Quick A. S., Risch P., Martin T., Hoose T., Thiel M., Helmer D., Kotz B. E. R. F., Risch P., Helmer D., Rapp B. E., Kotz F., Quick A. S., Martin T., Hoose T., Thiel M. (2021). Adv. Mater..

[cit13] Desponds A., Banyasz A., Chateau D., Tellal A., Venier A., Meille S., Montagnac G., Chevalier J., Andraud C., Baldeck P. L., Parola S., Desponds A., Banyasz A., Chateau D., Tellal A., Andraud C., Baldeck P. L., Parola S., Venier A., Meille S., Chevalier J. (2021). Small.

[cit14] Wen X., Zhang B., Wang W., Ye F., Yue S., Guo H., Gao G., Zhao Y., Fang Q., Nguyen C., Zhang X., Bao J., Robinson J. T., Ajayan P. M., Lou J. (2021). Nat. Mater..

[cit15] Aguirre-Cortés J. M., Moral-Rodríguez A. I., Bailón-García E., Davó-Quiñonero A., Pérez-Cadenas A. F., Carrasco-Marín F. (2023). Appl. Mater. Today.

[cit16] Ambrosi A., Pumera M. (2016). Chem. Soc. Rev..

[cit17] Bauer J., Hengsbach S., Tesari I., Schwaiger R., Kraft O. (2014). Proc. Natl. Acad. Sci. U. S. A..

[cit18] Jang D., Meza L. R., Greer F., Greer J. R. (2013). Nat. Mater..

[cit19] Xia H., Chen Q.-D., Sun H.-B., Fan H.-T., Guo L., Zhang T., Zhang Y.-L. (2010). Opt. Lett..

[cit20] Gailevičius D., Padolskytė V., Mikoliūnaitė L., Šakirzanovas S., Juodkazis S., Malinauskas M. (2019). Nanoscale Horiz..

[cit21] Yee D. W., Lifson M. L., Edwards B. W., Greer J. R., Yee D. W., Lifson M. L., Edwards B. W., Greer J. R. (2019). Adv. Mater..

[cit22] Winczewski J. P., Zeiler S., Gabel S., Maestre D., Merle B., Gardeniers J. G. E., Arce A. S. (2024). Mater. Des..

[cit23] Winczewski J. P., Dávila J. A., Herrera-Zaldívar M., Ruiz-Zepeda F., Córdova-Castr R. M., Pérez de la Vega C. R., Cabriel C., Izeddin I., Gardeniers H., Susarrey-Arce A. (2024). Adv. Mater..

[cit24] Grandcolas M., Lind A. (2022). Mater. Lett..

[cit25] Thomas N., Dionysiou D. D., Pillai S. C. (2021). J. Hazard. Mater..

[cit26] Chen X., Rong H., Ndagijimana P., Nkinahamira F., Kumar A., Guo D., Cui B. (2023). Results Eng..

[cit27] Imrich T., Zazpe R., Krýsová H., Paušová Š., Dvorak F., Rodriguez-Pereira J., Michalicka J., Man O., Macak J. M., Neumann-Spallart M., Krýsa J. (2021). J. Photochem. Photobiol., A.

[cit28] Yang X., Liu R., Du C., Dai P., Zheng Z., Wang D. (2014). ACS Appl. Mater. Interfaces.

[cit29] Habibi A., Rad K. N. (2019). Asia-Pac. J. Chem. Eng..

[cit30] Gendrot M., Jardot P., Delandre O., Boxberger M., Andreani J., Duflot I., Le Bideau M., Mosnier J., Fonta I., Hutter S., La Scola B., Pradines B. (2021). J. Clin. Med..

[cit31] Ramsay R. R., Dunford C., Gillman P. K. (2007). Br. J. Pharmacol..

[cit32] Snyder M., Gangadhara S., Brohl A. S., Ludlow S., Nanjappa S. (2017). Cancer Control.

[cit33] Castello M., Pais N., Nascimento E. De S. (2018). J. Pharm. Sci..

[cit34] Vo H. N. P., Le G. K., Nguyen T. M. H., Bui X. T., Nguyen K. H., Rene E. R., Vo T. D. H., Cao N. D. T., Mohan R. (2019). Chemosphere.

[cit35] Nunes B. (2020). Handb. Environ. Chem..

[cit36] Lindim C., van Gils J., Georgieva D., Mekenyan O., Cousins I. T. (2016). Sci. Total Environ..

[cit37] Adeleye A. S., Xue J., Zhao Y., Taylor A. A., Zenobio J. E., Sun Y., Han Z., Salawu O. A., Zhu Y. (2022). J. Hazard. Mater..

[cit38] Malik B., Majumder S., Lorenzi R., Perelshtein I., Ejgenberg M., Paleari A., Nessim G. D. (2022). Chempluschem.

[cit39] More S., Raut S., Premkumar S., Bhopale S., Bhoraskar S., More M., Mathe V. (2020). RSC Adv..

[cit40] Balan A. P., Radhakrishnan S., Woellner C. F., Sinha S. K., Deng L., Reyes C. D. L., Rao B. M., Paulose M., Neupane R., Apte A., Kochat V., Vajtai R., Harutyunyan A. R., Chu C. W., Costin G., Galvao D. S., Martí A. A., Van Aken P. A., Varghese O. K., Tiwary C. S., Iyer A. M. M. R., Ajayan P. M. (2018). Nat. Nanotechnol..

[cit41] Jakub Z., Meier M., Kraushofer F., Balajka J., Pavelec J., Schmid M., Franchini C., Diebold U., Parkinson G. S. (2021). Nat. Commun..

[cit42] Liu A., Liu J., Pan B., Zhang W. X. (2014). RSC Adv..

[cit43] Eyovge C., Deenen C. S., Ruiz-Zepeda F., Bartling S., Smirnov Y., Morales-Masis M., Susarrey-Arce A., Gardeniers H. (2021). ACS Appl. Nano Mater..

[cit44] Benkoula S., Sublemontier O., Patanen M., Nicolas C., Sirotti F., Naitabdi A., Gaie-Levrel F., Antonsson E., Aureau D., Ouf F. X., Wada S. I., Etcheberry A., Ueda K., Miron C. (2015). Sci. Rep..

[cit45] Murphy M., Walczak M. S., Hussain H., Acres M. J., Muryn C. A., Thomas A. G., Silikas N., Lindsay R. (2016). Surf. Sci..

[cit46] Briggs D. (1982). Surf. Interface Anal..

[cit47] Trenczek-Zajac A., Synowiec M., Zakrzewska K., Zazakowny K., Kowalski K., Dziedzic A., Radecka M. (2022). ACS Appl. Mater. Interfaces.

[cit48] Li C., Zhang Y., Qiu C., Yuan B., Zhang R., Li W., Jin H. (2023). Colloids Surf., A.

[cit49] Bernasconi R., Bellè U., Brigatti S., Diamanti M. V. (2024). Addit. Manuf..

[cit50] Kennedy A. J., McQueen A. D., Ballentine M. L., May L. R., Fernando B. M., Das A., Klaus K. L., Williams C. B., Bortner M. J. (2023). Chem. Eng. J..

[cit51] Clarizia L., Russo D., Di Somma I., Marotta R., Andreozzi R. (2017). Appl. Catal., B.

[cit52] Martín de Vidales M. J., Nieto-Márquez A., Morcuende D., Atanes E., Blaya F., Soriano E., Fernández-Martínez F. (2019). Catal. Today.

[cit53] Li L., Li J., Luo H., Li S., Yang J. (2022). Polymers.

[cit54] Viskadourakis Z., Sevastaki M., Kenanakis G. (2018). Appl. Phys. A: Mater. Sci. Process..

[cit55] Bansiddhi A., Panomsuwan G., Hussakan C., Htet T. L., Kandasamy B., Janbooranapinij K., Choophun N., Techapiesancharoenkij R., Pant H. R., Ang W. L., Jongprateep O. (2023). Top. Catal..

[cit56] Grandcolas M., Lind A. (2022). Mater. Lett..

[cit57] Grandcolas M., Oudin E. (2023). Environ. Chem. Lett..

[cit58] Cao X., Luo S., Liu C., Chen J. (2017). Adv. Powder Technol..

[cit59] Chau J. H. F., Lai C. W., Leo B. F., Juan J. C., Johan M. R. (2022). Catal. Commun..

[cit60] Ramos-Delgado N. A., Pino-Sandoval D. A., López-Velázquez K., Englezos C., Villanueva-Rodríguez M., Gracia-Pinilla M. A., Boscher N. D., Gardeniers H. J. G. E., Susarrey-Arce A. (2024). J. Photochem. Photobiol., A.

[cit61] Das A., Adak M. K. (2022). Appl. Surf. Sci. Adv..

[cit62] Hussain S., Aneggi E., Goi D. (2021). Environ. Chem. Lett..

[cit63] Guan Y., Zhao S., Li J., Deng X., Ma S., Zhang Y., Jiang B., Yao T., Xin B., Zhang J., Wu J. (2022). J. Colloid Interface Sci..

[cit64] Bansal P., Verma A. (2018). Mater. Sci. Eng., B.

