# Supplementary material for: Additive manufacturing of hollow connected networks for solar photo-Fenton-like catalysis
Source: RSC Sustain. 2024 Oct 16;2(12):3897–908. doi: 10.1039/d4su00312h (PMC11492987; doi:10.1039/d4su00312h)
Supplement: SU-002-D4SU00312H-s001 [file SU-002-D4SU00312H-s001.pdf]

Supplementary Information (SI) for RSC Sustainability.
This journal is © The Royal Society of Chemistry 2024

Additive Manufacturing of Hollow Connected Networks for Solar Photo-Fenton-Like Catalysis

Miguel Ángel Gracia-Pinilla[1,2,#,*], Norma Alicia Ramos-Delgado[1,3,#,*], Cristian Rosero-Arias[1,4], Remco Sanders[1], Stephan Bartling[5], Jędrzej Winczewski[1], Han Gardeniers[1], Arturo Susarrey-Arce[1,*]

[1]Mesoscale Chemical Systems, MESA+ Institute, University of Twente, PO Box 217, Enschede 7500 AE, the Netherlands

[2]Facultad de Ciencias Físico Matemáticas, Universidad Autónoma de Nuevo León, San Nicolás de los Garza, Nuevo León 66455, Mexico

[3]Centro de Investigación e Innovación Tecnológica, Cátedras CONACyT-Tecnológico Nacional de México/I.T. Nuevo León, Apodaca, Nuevo León, México

[4]School of Engineering and Sciences, Tecnologico de Monterrey, Eugenio Garza Sada 2501, Monterrey 64849, NL, Mexico

[5]Leibniz-Institut für Katalyse e.V., Albert-Einstein-Strasse 29 a, D-18059 Rostock, Germany

#These authors contributed equally.

1. Complimentary Scanning Electron Microscopy (SEM) images

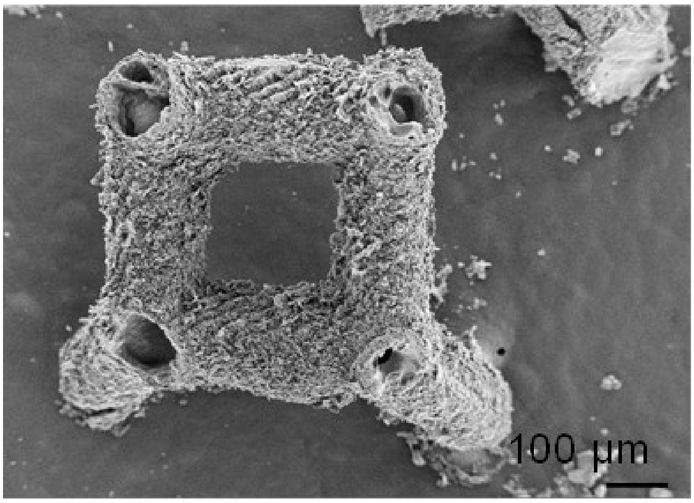

Figure S1. SEM image of broken 3DHMs composed of Al_2O_3 NPs.

SEM image of Al_2O_3 3D ceramic hollow microarchitecture printed using Phrozen aqua grey 4K photoresin is shown in **Figure S1**. Similar to TiO_2 3DHMs, SiO_2 has been found. SiO_2 traces are from the Phrozen aqua grey 4K resin.

2. Thermogravimetric analysis (TGA)

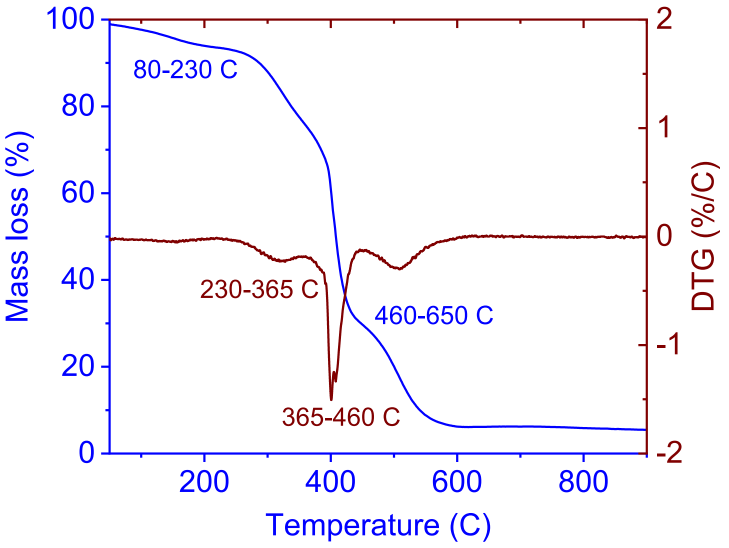

Figure S2. TGA of the TiO_2 3D hollow microarchitectures.

TGA analysis results present the mass losses (Mass loss % in blue), and differential thermogravimetric analysis (DTG %/C in magenta) shows four regions. Low-temperature region (80-230 °C) can be related to a low-temperature debinding stage. This could be related to solvents and small molecules that decompose within the low-temperature range. Diluent monomers and acrylic polymers might gasify. Within the same temperature range, the melted binder might achieve some fluidity. The process can be even more pronounced at higher temperatures (230-365 °C). At the higher temperature, close to 365 °C, decomposition of the printed structure, leading to carbon-like species, is achieved. More prominently, a mass loss in **Figure S2** is achieved around 365-460 C. The results align well with the size reduction of the 3D-printed microarchitecture (**Figure 1**). At higher temperatures (460-650 °C), the remaining carbon of the printed structure reacts to oxidized gaseous products to form hollow interconnected channels. At the high-temperature end (650 °C), most of the carbon-like remaining residues undergo intense calcination, creating the 3D printing hollow microarchitecture (**Figure 1**).

3. Complimentary X-ray photoelectron spectroscopy (XPS) data

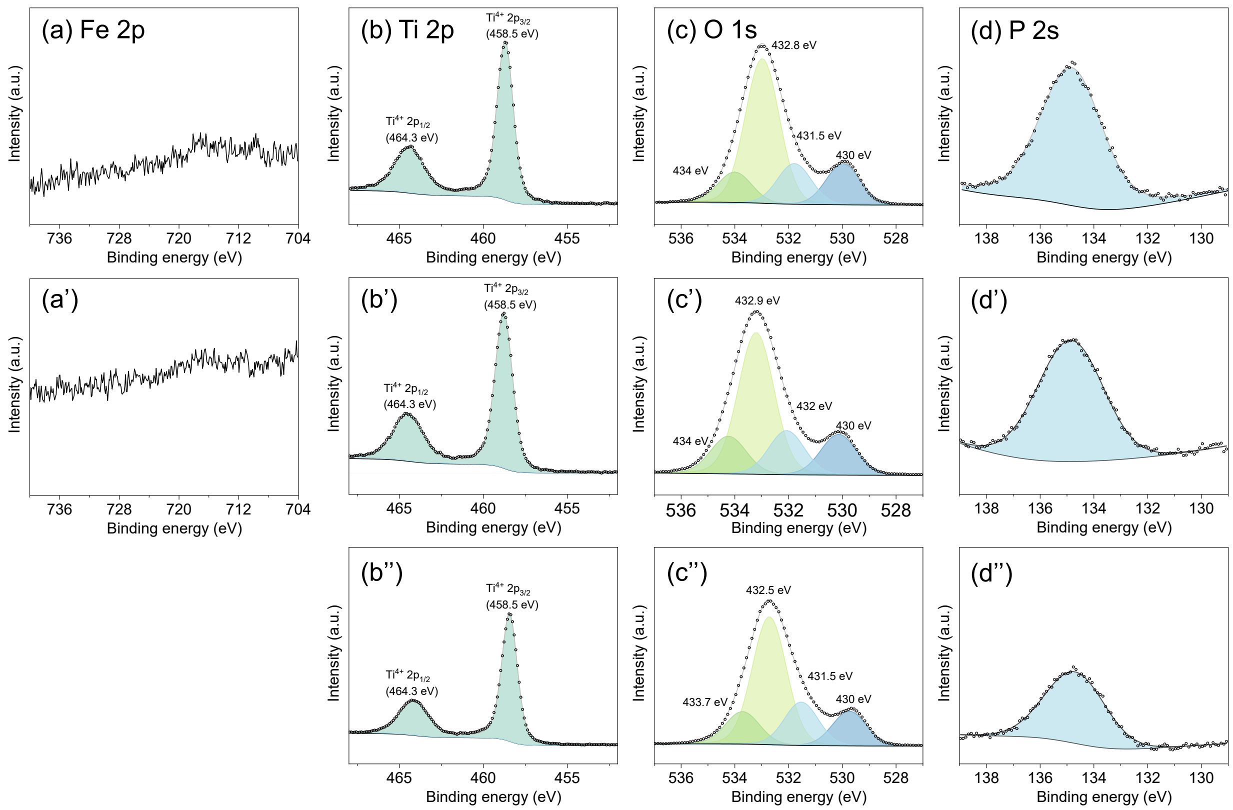

Figure S3. XPS core spectra for (a, a') Fe 2p, (b, b', b'') Ti 2p, (c, c', c'') O 1s, (d, d',d'') P 2s for (a, b, c, d) TiOFe2, (a', b', c', d') TiOFe0.5, and (b', c', d') TiO 3DHMs. Note that Fe 2p for the TiO_2 3DHM is not shown. No indications for Fe have been found in the general survey.

In **Figures 3Sa** and **3Sa'**, no Fe 2p has been resolved from the XPS analysis. The Ti 2p XPS in **Figures 3Sb**, **3Sb'**, and **3Sb''** displays two prominent peaks at 458.5 and 464.3 eV. The peaks are assigned to Ti^{4+} from TiO_2.[1] XPS O1s core level spectra exhibit the contribution at ca. ~530 eV attributed to bulk O in α- TiO_2 (**Figures 3Sc**, **3Sc'**, and **3Sc''**).[2] Surface-adsorbed H_2O and -OH as well as organic carbon contributions, are detected at higher binding energies (531-534 eV).[2] Interestingly, the P signature from the BAPO photoinitiator has been found in **Figures 3Sd**, **3Sd'**, and **3Sd''**,

4. Complimentary X-ray Powder Diffraction (XRD) patterns

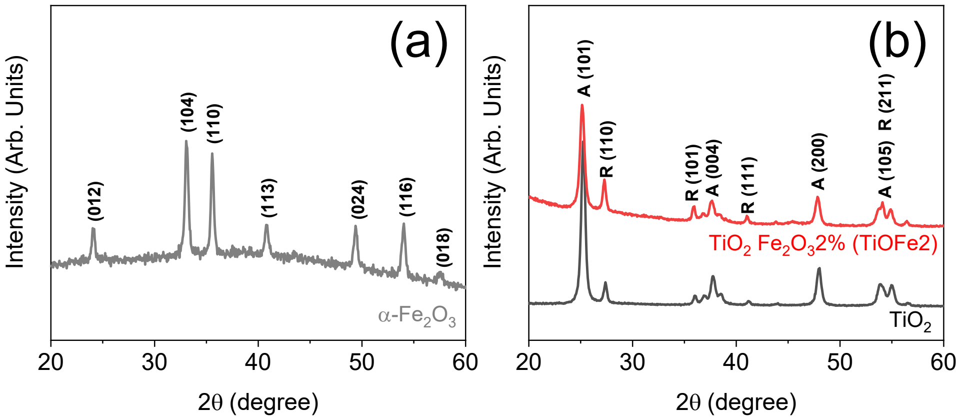

Figure S4. XRD of (a) α-Fe_2O_3 and (b) commercial TiO_2 and pulverized TiO_2 microarchitectures loaded with TiOFe2.

The XRD pattern in **Figure S4a** demonstrates the hematite crystallographic phase (α-Fe_2O_3) after synthesis. The prominent diffraction peaks observed are (012), (104), (110), (113), (024), (116), and (018), which are in agreement with the JCPDS file Card, No. 33-0664. **Figure S4b** reveals the XRD pattern of commercial TiO_2 (black), which is then compared with pulverized printed TiO_2 microarchitecture loaded with 2 wt.% α-Fe_2O_3 (red). The results from **Figure S4b** are assigned to the Anatase (A) and Rutile (R) crystallographic phases. The prominent diffraction peaks observed are for Anatase: (101), (004), (200), and (105), while for Rutile: (110), (101), and (111), which are in agreement with the JCPDS file Card, No. 00-021-1272, and JCPDS file Card, No. 01-070-7347. No α-Fe_2O_3 has been observed in **Figure S4b**.

5. Complimentary Ultraviolet-visible (UV-Vis) analysis

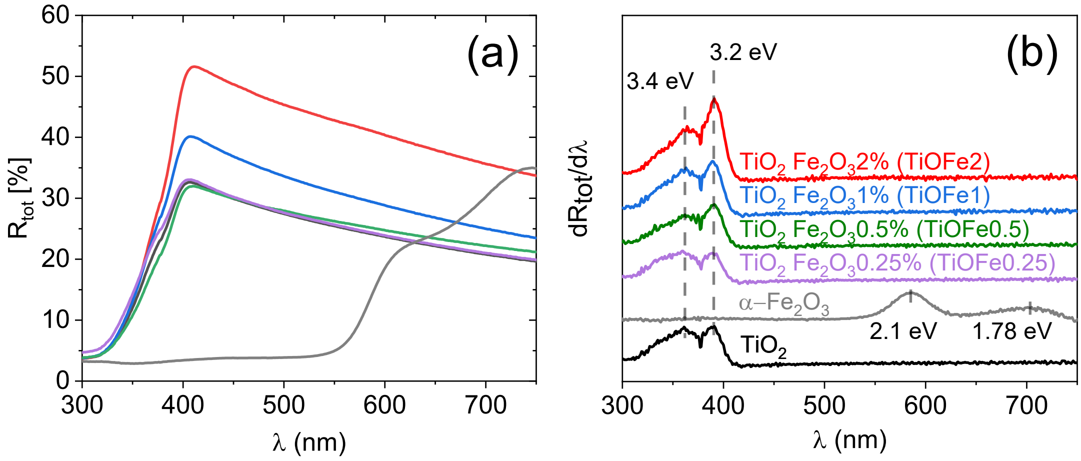

Figure S5. (a) spectral dependence of the total optical reflectance R_{tot} of TiO_2 microarchitectures loaded with 2 wt.% (red), 1 wt.% (blue), 0.5 wt.% (green), and 0.25 wt.% (violet) of α-Fe_2O_3. (b) first derivate spectra of TiO_2 microarchitectures loaded with 2 wt.% (red), 1 wt.% (blue), 0.5 wt.% (green), and 0.25 wt.% (violet) of α-Fe_2O_3. TiO_2 (black) and α-Fe_2O_3 (grey) are used for comparison.

6. Energy band diagram

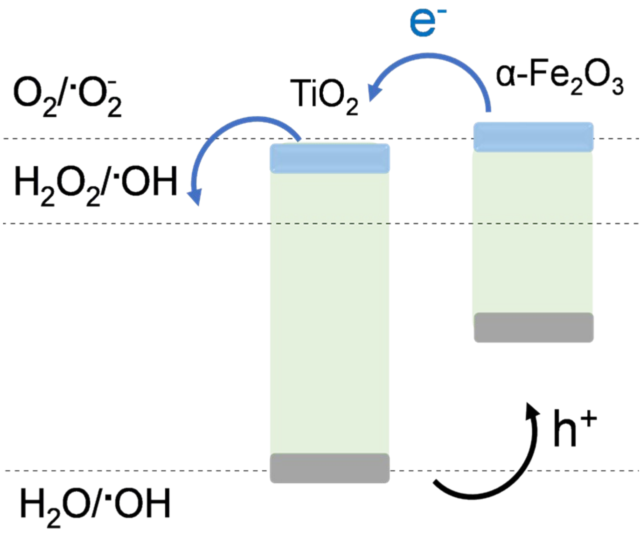

Figure S6. Energy band diagrams using the VB and Eg of printed TiO_2 and synthesized α-Fe_2O_3.

The mechanism is shown in **Figure S7** and suggests photoelectron (e^-) generation at the CB and holes (h^+) at the VB, aligning with the proposed mechanism in **Figure 5d**.

7. Adsorption-degradation synergistic effect

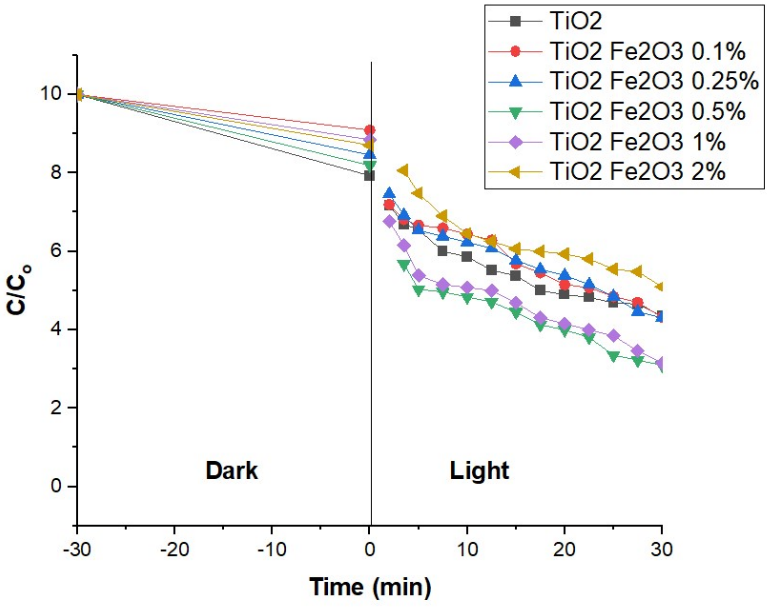

Figure S7. MB adsorption-degradation synergistic effect (-30 to 30 minutes) time for 3D microarchitectures composed of TiO_2 and TiO_2 loaded with 2, 1, 0.5, 0.25 wt.% of α-Fe_2O_3.

8. Previous work

Table S1. Comparative table of 3D printing method and reported MB photodegradation

3D Additive Method	NPs Size (nm)	MB (ppm)	Photo-degradation (%) / Time (h)	Light Source (W)	Size 3D (mm)	Ref
1% TiO_2/LDPE Floating structures by Fused Filament Fabrication (FFF)	<25	10	15 / 2 h	UV lamp	Square mesh (25x25x2)	[3]
6% TiO_2 Chitin / Cellulose composites by Printing ink	<20	50	90 / 10 h	UV lamp (300)	Cube (10x10x10)	[4]
20% TiO_2 polystyrene filament by Fused Deposition Modeling (FDM)	<25	20	95 / 1h	UV lamp (125)	16 Cilinders (10x10x10)	[5]
5% TiO_2 / SiO / polymers by Stereolithography (SLA)	<25	5	5 (75 Adsorption x 24 hr) / 8 h	UV lamp (6)	hexagonal (5x5x5)	[6]
2% TiO_2 / polyamide by Selective Laser Sintering (SLS)	<25	10	65 / 3 h	UV lamp (200)	Lattice structure (54x54x50)	[7]
10% TiO_2 / Polyacrylonitrile by Electrospinning	<25	10	55 / 3 h	UV Lamp (200)	Nanofiber (150-400nm)	[8]
Nanoparticles	<70	10	70 / 2h	Vis Lamp (500)	Nanoparticles (50-70nm)	[9]
**4% TiO_2 in commercial resin by Digital Light Processing (DLP)**	**<25**	**10**	**95 / 3 h**	**UV Led (0.06)**	**Cube (Truncated Octahedron) (5x5x5)**	**our work**

References

[1] C. Eyovge, C.S. Deenen, F. Ruiz-Zepeda, S. Bartling, Y. Smirnov, M. Morales-Masis, A. Susarrey-Arce, H. Gardeniers, Color Tuning of Electrochromic TiO2Nanofibrous Layers Loaded with Metal and Metal Oxide Nanoparticles for Smart Colored Windows, ACS Appl Nano Mater 4 (2021) 8600–8610. https://doi.org/10.1021/acsanm.1c02231.

[2] S. Benkoula, O. Sublemontier, M. Patanen, C. Nicolas, F. Sirotti, A. Naitabdi, F. Gaie-Levrel, E. Antonsson, D. Aureau, F.X. Ouf, S.I. Wada, A. Etcheberry, K. Ueda, C. Miron, Water adsorption on TiO2 surfaces probed by soft X-ray spectroscopies: bulk materials vs. isolated nanoparticles, Scientific Reports 2015 5:1 5 (2015) 1–11. https://doi.org/10.1038/srep15088.

[3] M.J. Martín de Vidales, A. Nieto-Márquez, D. Morcuende, E. Atanes, F. Blaya, E. Soriano, F. Fernández-Martínez, 3D printed floating photocatalysts for wastewater treatment, Catal Today 328 (2019) 157–163. https://doi.org/10.1016/j.cattod.2019.01.074.

[4] L. Li, J. Li, H. Luo, S. Li, J. Yang, Physicochemical and Photocatalytic Properties of 3D-Printed TiO2/Chitin/Cellulose Composite with Ordered Porous Structures, Polymers (Basel) 14 (2022). https://doi.org/10.3390/polym14245435.

[5] Z. Viskadourakis, M. Sevastaki, G. Kenanakis, 3D structured nanocomposites by FDM process: a novel approach for large-scale photocatalytic applications, Appl Phys A Mater Sci Process 124 (2018) 1–8. https://doi.org/10.1007/S00339-018-2014-6/METRICS.

[6] A. Bansiddhi, G. Panomsuwan, C. Hussakan, T.L. Htet, B. Kandasamy, K. Janbooranapinij, N. Choophun, R. Techapiesancharoenkij, H.R. Pant, W.L. Ang, O. Jongprateep, Ecofriendly 3D Printed TiO2/SiO2/Polymer Scaffolds for Dye Removal, Top Catal 66 (2023) 1662–1673. https://doi.org/10.1007/s11244-023-01864-x.

[7] M. Grandcolas, A. Lind, 3D-printed polyamide structures coated with TiO2 nanoparticles, towards a 360-degree rotating photocatalytic reactor, Mater Lett 307 (2022). https://doi.org/10.1016/j.matlet.2021.131044.

[8] M. Grandcolas, E. Oudin, Enhanced photocatalytic activity of electrospun TiO2/polyacrylonitrile membranes in a crossflow reactor using dual lights, Environ Chem Lett 21 (2023) 633–638. https://doi.org/10.1007/s10311-022-01553-3.

[9] X. Cao, S. Luo, C. Liu, J. Chen, Synthesis of Bentonite-Supported Fe2O3-Doped TiO2 superstructures for highly promoted photocatalytic activity and recyclability, Advanced Powder Technology 28 (2017) 993–999. https://doi.org/10.1016/j.apt.2017.01.003.
